# Ameliorative effects of *Toxocara canis* recombinant C-type lectin on atopic dermatitis in a mouse model

**DOI:** 10.1016/j.jacig.2026.100756

**Published:** 2026-06-30

**Authors:** Hamidreza Jahani, Shahram Jamshidi, Fateme Jalousian, Sara Shokrpoor, Maryam Nourizadeh, Seyed Hossein Hosseini, Parmida Malekzadeh, Zahra Boluki

**Affiliations:** a Department of Internal Medicine, Faculty of Veterinary Medicine, University of Tehran, Tehran, Iran; b Department of Parasitology, Faculty of Veterinary Medicine, University of Tehran, Tehran, Iran; c Department of Pathology and Clinical Pathology, Faculty of Veterinary Medicine, University of Tehran, Tehran, Iran; d Department of Immunology, Asthma and Allergy Research Institute, Tehran University of Mepical Sciences, Tehran, Iran; e Knowledge Utilization Research Center, Tehran University of Mepical Sciences, Tehran, Iran

**Keywords:** Atopic dermatitis score, 2,4-dinitrochlorobenzene (DNCB), histopathology, IgE ELISA assay, immunomodulation, recombinant protein therapy

## Abstract

**Background:**

Atopic dermatitis (AD) is a chronic inflammatory skin disease marked by severe itching and eczematous lesions, primarily driven by immune system dysregulation. Current treatments largely focus on symptom relief rather than addressing underlying immune dysfunction. The parasitic nematode *Toxocara canis* secretes excretory-secretory proteins, among which C-type lectin (CTL) has notable immunomodulatory properties that may offer a novel therapeutic approach for AD.

**Objective:**

We sought to evaluate the therapeutic potential of *T canis* recombinant CTL (rCTL) in a BALB/c mouse model of AD.

**Methods:**

Thirty-five mice were divided into 7 groups; all except from the normal control group were sensitized and challenged with 2,4-dinitrochlorobenzene to induce AD-like symptoms. Treatment groups received rCTL, topical or injected dexamethasone, or bacterial lysate. The prevention group was pretreated with rCTL (subcutaneously and intraperitoneally) before 2,4-dinitrochlorobenzene exposure. Dermatitis severity was assessed biweekly, and pruritus was quantified through blinded video analysis of scratching behavior. Serum IgE levels were measured via ELISA, and histopathologic analyses examined epidermal and dermal changes.

**Results:**

*T canis* rCTL significantly reduced dermatitis severity, pruritic behavior, and serum IgE compared with controls, including in dexamethasone-treated groups (*P* < .05). Histology revealed reduced epidermal thickening and decreased inflammatory cell infiltration in rCTL-treated mice, indicating effective suppression of allergic inflammation. The prevention achieved by rCTL was modest and not highly effective.

**Conclusions:**

*T canis* rCTL shows promising immunomodulatory effects, improving both clinical and histopathologic features of AD in mice. These findings highlight rCTL’s potential as a novel immunotherapeutic agent, warranting further mechanistic and long-term studies to confirm clinical applicability.

Atopic dermatitis (AD) is a chronic inflammatory skin condition characterized by intense itching, redness, and lesions, significantly affecting the quality of life for affected individuals. The pathogenesis of AD involves complex interactions between genetic predispositions, environmental factors, and immune responses, particularly those mediated by T_H_2 cells and the subsequent elevation of IgE levels. T_H_2 cytokines, such as IL-4, IL-5, and IL-13, contribute to the inflammation and immune dysregulation observed in AD, whereas increased IgE levels facilitate allergic responses.^1^

AD affects approximately 10% to 20% of children and 2% to 10% of adults globally, with a prevalence of about 2.6%.^2^ A systematic review and meta-analysis published in 2023 analyzed 30 studies on pediatric AD in Iran from the past decades. The overall prevalence of AD was found to be approximately 7.4%, with girls showing a prevalence rate of about 8.5% and boys about 8.1%. This study concluded that although the prevalence in Iran is considerable, it is generally lower than that reported in other countries.^2^ The recent increase in the prevalence of AD globally has been linked to multiple factors, including urbanization, changes in diet, and heightened exposure to allergens. Interestingly, studies have shown that early exposure to pets, especially dogs, may reduce the prevalence of AD in children, indicating a protective effect of certain environmental factors on disease development.^3^

Canine atopic dermatitis (CAD) is another significant health concern, with prevalence rates ranging from 3% to 15%, and studies suggest that up to 25% of dogs treated at veterinary clinics for dermatologic issues are diagnosed with CAD.^4^ CAD shares similar immunologic and clinical features with human AD, making it an ideal model for understanding the disease mechanisms and testing therapeutic interventions. In addition, feline AD, although less studied, is a common dermatologic issue, with prevalence estimates of about 12.5%.^4^ The shared features across species highlight the potential for cross-species therapeutic strategies.

The treatment of AD in both humans and animals requires a multifaceted approach. In dogs, management focuses on controlling acute flares, suppressing chronic inflammation, restoring the skin barrier, and allergen desensitization. Key treatments for canine AD include allergen-specific immunotherapy, oclacitinib (a Janus kinase 1 [JAK1] inhibitor), modified cyclosporine, and oral corticosteroids.^5^ Similarly, human AD management centers on barrier restoration and inflammation reduction through topical therapies and systemic medications such as cyclosporine for severe cases.^6^ The introduction of JAK inhibitors, including oclacitinib in veterinary medicine and topical ruxolitinib, oral abrocitinib, and upadacitinib in human medicine, has created new therapeutic possibilities for inflammatory skin conditions across both species.^7^^,^^8^

Current therapeutic options for managing AD focus on alleviating symptoms and controlling inflammation. Topical corticosteroids remain the cornerstone of treatment but are associated with side effects such as skin atrophy and increased susceptibility to infections. Systemic corticosteroids, although effective in severe cases, carry risks of adrenal suppression, growth retardation in children, and rebound flaring on discontinuation. Newer targeted therapies, including biologics such as dupilumab and JAK inhibitors, have shown improved efficacy but are not without drawbacks. JAK inhibitors such as oclacitinib have shown efficacy in managing AD by selectively inhibiting the JAK1 pathway, which plays a key role in proinflammatory cytokines such as IL-4, IL-5, and IL-13 that contribute to the pathogenesis of AD. Oclacitinib is approved for the treatment of allergic dermatitis in dogs and has anecdotal evidence of symptom relief in humans.^9^ JAK inhibitors, for example, carry risks of adverse events, including infections, cytopenias, and hypertension.^8^^,^^9^ These therapies are also expensive, limiting accessibility for many patients. Systemic immunosuppressants, such as cyclosporine and mycophenolate mofetil, show efficacy but are limited by rapid relapse on cessation and concerns over long-term safety.^9^ Despite these advances, there remains a pressing need for novel treatments that can effectively address the underlying immune dysregulation in AD while offering improved safety and durability of response.^10^ One promising avenue for novel treatments is the immunomodulatory properties of *Toxocara canis*, a parasitic nematode commonly found in dogs. Recent studies have highlighted the potential of recombinant C-type lectin (rCTL) derived from *T canis* to modulate immune responses.^10^ rCTL enhances regulatory T-cell populations, which are crucial for maintaining immune homeostasis and preventing excessive inflammation.^11^ Previous studies have also demonstrated that rCTL can significantly reduce disability scores in the experimental autoimmune encephalomyelitis (EAE) mouse model, which mimics the inflammatory processes seen in multiple sclerosis.^12^ On the basis of these findings, it is hypothesized that rCTL could similarly modulate immune responses in AD.

The present study aimed to evaluate the potential of rCTL derived from *T canis* as a therapeutic agent for AD. Using a BALB/c mouse model of AD induced by 2,4-dinitrochlorobenzene (DNCB), this study assesses the impact of rCTL on clinical signs of AD, immune response modulation, and associated behaviors. By targeting both the symptoms and the underlying immune dysregulation of AD, rCTL may offer a novel therapeutic strategy for managing this chronic condition.

## Methods

### Expression, extraction, and purification of rCTL

The expression, extraction, and purification of *T canis* rCTL were performed in the Molecular Parasitology Laboratory, Department of Parasitology, Faculty of Veterinary Medicine, University of Tehran (Tehran, Iran), following the methodology described by Malekzadeh et al.^13^ The open reading frame that encodes the CTL protein of *T canis* was retrieved from GenBank (accession no. KU852582). Following codon optimization, additional sequences for enzymatic restriction sites and a stop codon were incorporated into the original sequence. The recombinant plasmid pET32a, containing this modified sequence, was synthesized by GENERAY Co (Shanghai, China). To produce the recombinant protein, the plasmid was introduced into competent *Escherichia coli* BL21 (DE3) bacteria (Bon Yakhteh Co, Tehran, Iran) using a heat shock method with a 0.1 mol solution of calcium chloride and magnesium chloride (CaCl_2_-MgCl_2_). The successful transformation of the plasmid into the competent bacteria was confirmed through bacterial growth assays on Luria-Bertani agar plates (Merck) supplemented with ampicillin at a concentration of 100 mg/mL, along with enzyme restriction analysis using BamHI and EcoRI (Thermo Fisher Scientific) and colony PCR.

**Fig 1:**
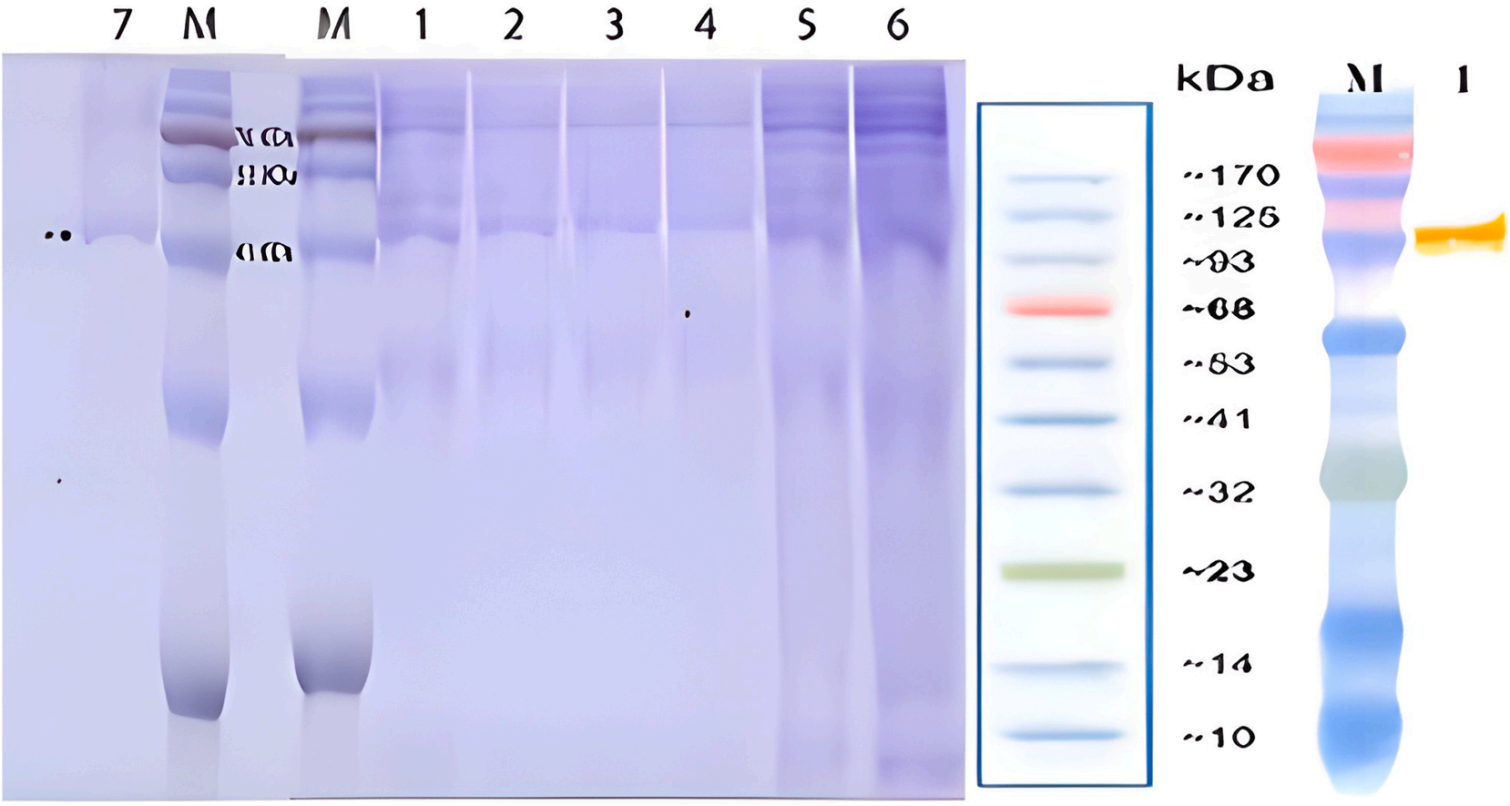
SDS-PAGE analysis was conducted on a 12% acrylamide gel to assess His-6–tagged rCTL from *T canis*. A prestained protein ladder (10-170 kDa) served as a molecular weight marker. Various bacterial lysates were analyzed postinduction with isopropyl β-d-1-thiogalactopyranoside, revealing a band corresponding to the 41-kDa rCTL. Western blotting confirmed the presence of the His-tagged rCTL using an mAb at a 1:1000 dilution.

Induction of recombinant protein expression was achieved by adding isopropyl β-d-1-thiogalactopyranoside (Sigma) at a concentration of 1 mM and incubating the cultures at 37°C for 4 hours. Following induction, the resulting cell pellet was subjected to sonication at a frequency of 40 kHz for 30 seconds (Hielscher, Chicago, Ill). Purification of the recombinant protein was performed using the QIAGEN Ni-NTA spin kit (QIAGEN) under denaturing conditions, following the manufacturer’s protocol, with 8 mol urea (Merck) and lysozyme (DNA Biotech, Iran). The presence and purity of the recombinant proteins were analyzed using 12% SDS-PAGE (Merck). The proteins separated by SDS-PAGE were then transferred to a 0.45-μm nitrocellulose membrane (Bio-Rad, Munich, Germany) using TRIS-glycine buffer (25 mM TRIS-glysin, 192 mM glysin, and pH 8.3) (Merck) containing 0.05% Tween at 45 V for 5 hours. Western blotting was conducted using an anti–6-histidine antibody (Sigma-Aldrich, Darmstadt, Germany) diluted to 1:1000 and visualized with 3,3'-diaminobenzidine (Sigma-Aldrich) (Fig 1).

### Animals

Thirty-five 5-week-old male BALB/c mice were acquired from the Center of Experimental and Comparison Studies, Iran University of Medical Sciences (Tehran, Iran). The mice were housed individually in ventilated cages under specific pathogen-free conditions in the animal laboratory’s breeding center at the Faculty of Veterinary Medicine, University of Tehran. Special attention was also given to ensuring their skin was intact, lesion-free, and without any other pathologies. The mice were provided with continuous access to clean, fresh tap water and food for 24 hours a day. The laboratory conditions were controlled, maintaining a temperature range of 22°C to 25°C and relative humidity between 45% and 65%. In addition, a 12-hour light-dark cycle was implemented in accordance with the Guide for the Care and Use of Laboratory Animals released by the National Research Council (2011). This experiment was approved by the Iran National Committee for Ethics in Biomedical Research.

After a 1-week adaptation period, the sample size for each group of BALB/c mice was calculated on the basis of a 95% confidence level, a 5% margin of error (Zα/2 = 1.96), 80% statistical power (Zβ = 0.8), a variance (*δ*) of 0.8, and a minimum detectable difference between group means (μ1 − μ2) of 1.5.^14^ Attrition of 10% was anticipated and mice were divided into the following 7 groups (n = 5 mice/group), each with a similar weight of approximately 19 ± 1 g: (1) NC (normal control): vehicle applied topically, no dermatitis induction; (2) DC (DNCB control): dermatitis induced with DNCB, followed by vehicle treatment; (3) tDEX (topical dexamethasone): dermatitis induced with DNCB, treated with topical dexamethasone; (4) rCTL: dermatitis induced with DNCB, treated with rCTL administered subcutaneously and intraperitoneally; (5) iDEX (injected dexamethasone): dermatitis induced with DNCB, treated with dexamethasone administered subcutaneously and intramuscularly; (6) BL (bacterial lysate): dermatitis induced with DNCB, treated with *E coli* BL21 (DE3) lysate administered both subcutaneously and intramuscularly; and (7) PG (prevention group): rCTL treatment (administered subcutaneously and intraperitoneally) before DNCB-induced dermatitis.

### Induction of AD using DNCB in mice

AD was induced in mice using DNCB (Merck, Darmstadt, Germany). Mice were sensitized with DNCB applied to their skin on day 1 and subsequently challenged with DNCB every 3 days for a total of 4 weeks.

A day before the experiment, the dorsal skin of the mice was shaved (∼4 cm^2^) to prepare for the DNCB challenge. To induce AD-like skin lesions, solutions of 1% and 0.2% DNCB were prepared by dissolving in a mixture of acetone and olive oil (3:1). On the first day, 150 μL of the 1% DNCB solution was applied to the dorsal skin, whereas 20 μL was administered to the face and the concave surface of the left ear pinna. On day 5, the same volume of the 0.2% DNCB solution was applied to these areas, and this procedure was repeated 3 times a week for 4 weeks (on days 1, 5, 7, 9, 12, 14, 16, 19, 21, 23, 26, 28, 30, and 33). Starting on day 7, an hour after DNCB application, rCTL was injected both subcutaneously and intraperitoneally at a dose of 50 μg per site in the rCTL group. This dose was selected in accordance with earlier research conducted by Shahbakhsh et al,^15^ which determined the optimal, nontoxic effective dose of the rCTL. In the tDEX group, a topical application of 200 μL of a 0.1% dexamethasone solution prepared in the same vehicle as DNCB was administered to the dorsal skin and 20 μL to the face and the ear pinna. To evaluate the preventive effect of rCTL, mice in the PG group first received 12 administrations of rCTL injections intraperitoneally and subcutaneously before undergoing the same DNCB challenge as other groups. This group received 6 100-μg rCTL injections both intraperitoneally and subcutaneously over 2 weeks (3 days each week). According to previous studies by Malekzade et al,^13^ Shahbakhsh et al,^15^ and Etebar et al,^16^ a higher dose of rCTL is recommended for prevention than for treatment. The iDEX group followed a similar schedule for dexamethasone administration as the tDEX group but received a dose of 10 mg/kg intramuscularly. In the BL group, bacterial lysate was injected subcutaneously and intraperitoneally in amounts equivalent to rCTL at the same intervals. Finally, the NC group received only vehicle (acetone:olive oil; 3:1) topically and PBS injections. The number of mice in all groups was the same. Mice with significantly lower or higher pruritus behaviors were excluded before the start of the experiment—specifically, before dermatitis induction and after the mice had adapted to the laboratory environment—to ensure consistent baseline conditions across groups. At the end of the study, all mice were anesthetized and euthanized using 2% isoflurane; subsequently, skin samples were taken and blood samples were collected from the heart. The sera extracted from blood samples were stored at −80°C until analysis for determining IgE levels (Tables I and II).

**Table I:** The mean of dermatitis scores in the different groups over 5 wk∗

Groups	W1S1	W1S2	W2S1	W2S2	W3S1	W3S2	W4S1	W4S2	W5S1
Only vehicle (n = 5)	0	0	0	0	0	0	0	0	0
Only DNCB (n = 5)	11 ± 0.5	9 ± 1.0	8 ± 0.9	8 ± 0.8	8 ± 0.5	8 ± 0.4	7 ± 0.5	7 ± 0.5	8 ± 0.5
DNCB + topical dexamethasone (n = 5)	9 ± 1.3	8.5 ± 1.9	7 ± 2.9	6.5 ± 2.5	5 ± 2.0	4.5 ± 1.9	4 ± 1.7	4.5 ± 2.3	4.5 ± 3.0
DNCB + rCTL (n = 5)	10 ± 0.7	6 ± 2.3	6 ± 2.1	4 ± 1.6	4 ± 1.9	4 ± 1.5	5 ± 0.8	4 ± 1.5	3 ± 1.1
rCTL + DNCB (n = 5)	6 ± 0.8	7 ± 1.1	8 ± 1.3	7 ± 1.0	7 ± 1.0	5 ± 1.1	5 ± 1.7	5 ± 0.8	5 ± 0.8
DNCB + BL (n = 5)	9 ± 1.4	7 ± 1.1	8 ± 2.7	6 ± 1.5	6 ± 1.5	6 ± 0.8	7 ± 1.3	7 ± 1.3	8 ± 1.1
DNCB + injected dexamethasone (n = 5)	8.5 ± 1.3	7 ± 1.5	8 ± 1.1	7 ± 0.9	7 ± 1.0	7 ± 1.1	6 ± 0.7	5 ± 0.5	5 ± 0.7

Data are presented as mean ± SD. Scoring was conducted over a 5-wk period: twice weekly for the first 4 wk and once in the fifth week.

*W1S1-W5S1*, Week 1 scoring 1 through week 5 scoring 1.

∗ Each group was evaluated twice a week.

**Table II:** Pairwise comparison of dermatitis scores between groups over 5 wk

Groups	Only vehicle	Only DNCB	DNCB + topical dexamethasone	DNCB + rCTL	rCTL + DNCB	DNCB + BL	DNCB + injected dexamethasone
Only vehicle (n = 5)	—	<.05 (−8.9 to −7.3)	<.05 (−6.8 to −5.2)	<.05 (−5.7 to −4.2)	<.05 (−6.7 to −5.1)	<.05 (−7.9 to −6.4)	<.05 (−7.4 to −5.9)
Only DNCB (n = 5)	<.05 (7.3 to 8.9)	—	<.05 (1.3 to 2.9)	<.05 (2.3 to 3.9)	<.05 (1.4 to 2.9)	<.05 (0.17 to 1.7)	<.05 (0.74 to 2.2)
DNCB + topical dexamethasone (n = 5)	<.05 (5.2 to 6.8)	<.05 (−2.9 to −1.3)	—	<.05 (0.18 to 1.8)	.859 (−0.7 to 0.89)	<.05 (−1.9 to 0.34)	.11 (−1.4 to 0.15)
DNCB + rCTL (n = 5)	<.05 (4.2 to 5.7)	<.05 (−3.9 to −2.3)	<.05 (−1.8 to −0.18)	—	<.05 (−1.7 to −0.15)	<.05 (−2.9 to −1.4)	<.05 (−2.3 to −0.9)
rCTL + DNCB (n = 5)	<.05 (5.1 to 6.7)	.859 (−0.89 to 0.75)	<.05 (−2.9 to −1.4)	<.05 (0.15 to 1.7)	—	.06 (−1.4 to 0.03)	<.05 (0.4 to 2.0)
DNCB + BL (n = 5)	<.05 (6.0 to 7.9)	.153 (−0.2 to 1.2)	<.05 (0.34 to 1.9)	<.05 (1.4 to 2.9)	.06 (0.03 to −1.4)	—	<.05 (0.46 to 2.0)

Data are presented as *P* value (95% CI).

### Clinical sign scoring

#### Dermatitis scoring methodology

During the study, photographs of the dorsal skin, ears, and faces of the mice were taken twice a week using a digital camera. The scoring system for lesion severity was defined according to the method detailed by Jang et al.^17^ The scoring criteria included 4 parameters: erythema/hemorrhage, scarring, edema, and scaling/dryness. Each criterion was scored as follows: severe lesions: score of 3; moderate lesions: score of 2; mild lesions: score of 1; and no lesions: score of 0. Each parameter was scored up to 3 and the total dermatitis score was calculated by averaging the scores from face, ear, and dorsal skin, resulting in a maximum possible score of 12 per mouse, which indicated the most severe lesion presentation. All evaluations were conducted by a single blind investigator to ensure consistency in scoring.

#### Scratching behavior assessment

Once a week, a 10-minute video was recorded for each mouse to evaluate scratching behavior. Scratching events were defined as any instance in which a hind limb or forelimb made contact with the dorsal skin, face, or left ear of the mouse. Event quantification was performed by an observer blinded to the experimental groups. Each contact was counted as 1 scratching event, and the total number of events was calculated over the 10-minute observation period. Before the commencement of the study, baseline scratching behavior was assessed for each mouse. Mice exhibiting significantly higher or lower levels of scratching compared with their peers were excluded from the experiment to ensure uniformity in the assessment.

### Histopathology of mice skin

The pathology study involved assessing the effects of dermatitis induction on tissue samples collected from experimental mice. This work was carried out in the Pathology Department at the Faculty of Veterinary Medicine, University of Tehran. The pathologists were blinded to the sample identities. After the treatment period, mice were euthanized on day 21, and dorsal skin samples were collected from both the treated and control groups for histopathologic analysis. Collected skin samples were fixed in formalin, embedded in paraffin, and sectioned into 5-μm thick slices. The sections were stained with hematoxylin and eosin (H&E) and also toluidine blue for general histologic examination.

For each experimental group, 20 random H&E-stained 100× magnification microscope fields were assessed by measuring epidermal and dermal thickness, eosinophil number, and overall histopathology. The histopathology evaluations used a standardized scoring system assessing morphologic criteria including inflammatory cell infiltration and epidermal hyperplasia.^18^

### IgE ELISA assay

Quantitative determination of IgE levels was conducted using a commercially available mouse IgE ELISA kit (Biotime, Xiamen, China) in the Serology Laboratory of the Department of Parasitology, Faculty of Veterinary Medicine, University of Tehran. The assay was performed according to the manufacturer’s instructions by an investigator who was blinded to the experimental groups.

### Statistical analysis

In this study, ANOVA was used to compare the mean severity scores, IgE levels, and pathology markers across different groups. Statistical analyses were performed using SPSS software version 23, with a significance level set at .05.

## Results

The findings of the study indicated that the mean dermatitis score at the fifth week was significantly lower in the group treated with rCTL after DNCB induction (3.4 ± 1.14), as well as in the groups treated with topical and injected dexamethasone (4.6 ± 0.89), compared with the other groups (Tables I and II).

The comparison of dermatitis scores between the 2 groups treated with rCTL—one group receiving treatment before DNCB induction and the other after—revealed that the group treated with rCTL after DNCB induction had a significantly lower dermatitis score (Table I). This group also exhibited a significantly reduced pruritus behavior score (Tables III and IV). In addition, the IgE level in this group was significantly reduced, measuring at 18.76 ± 3.8 IU/mL (Table V). The mean twice-weekly dermatitis scores from different groups during the 5-week study were significant, except between the tDEX group and the PG and iDEX groups, between the PG and iDEX groups, and between the BL and iDEX groups (Table V). In week 5, only 1 injection was administered, and in the second round, the mice were euthanized.

**Table III:** The mean of pruritus behavior in the different groups over 5 wk∗

Groups	PBW1	PBW2	PBW3	PBW4	PBW5
Only vehicle (n = 5)	3 ± 0.7	4 ± 0.8	3 ± 0.7	3 ± 0.7	3 ± 1
Only DNCB (n = 5)	3 ± 1.2	9 ± 1.9	16 ± 1.5	19 ± 1.8	20 ± 0.8
DNCB + topical dexamethasone (n = 5)	3 ± 1.2	8 ± 1.4	12 ± 1.6	11 ± 1.6	11 ± 0.9
DNCB + rCTL (n = 5)	3 ± 0.5	5 ± 1	6 ± 1.5	7 ± 1.5	7 ± 1.3
rCTL + DNCB (n = 5)	4 ± 1	10 ± 2.1	15 ± 1.2	18 ± 2.7	19 ± 1.5
DNCB + BL (n = 5)	3 ± 0.7	11 ± 2.2	14 ± 1.2	17 ± 3.6	20 ± 2.1
DNCB + injected dexamethasone (n = 5)	4 ± 0.8	7 ± 1.4	11 ± 1.2	10 ± 1.4	9.5 ± 1.2

Data are presented as mean ± SD.

*PBW1-PBW5*, pruritus behavior scores in weeks 1 to 5.

∗ Each group was evaluated once a week.

**Table IV:** Pairwise comparison of pruritus behavior between groups over 5 wk

Groups	Only vehicle	Only DNCB	DNCB + t dexamethasone	DNCB + rCTL	rCTL + DNCB	DNCB + BL	DNCB + injected dexamethasone
Only vehicle (n = 5)	—	<.05 (−11.2 to −9.4)	<.05 (−6.8 to −4.9)	<.05 (−3.2 to −1.4)	<.05 (−10.2 to −9.1)	<.05 (−10.7 to −8.9)	<.05 (−6.0 to −4.3)
Only DNCB (n = 5)	<.05 (9.4 to 11.2)	—	<.05 (3.4 to 5.3)	<.05 (7.1 to 8.8)	.585 (0.64 to 1.1)	.319 (−0.44 to 1.3)	<.05 (−6.4 to 1.1)
DNCB + topical dexamethasone (n = 5)	<.05 (4.9 to 6.8)	<.05 (−5.3 to −3.4)	—	<.05 (2.6 to 4.5)	<.05 (−5.0 to−3.1)	<.05 (−4.8 to−2.9)	.125 (−0.2 to 1.6)
DNCB + rCTL (n = 5)	<.05 (1.4 to 3.2)	<.05 (−8.8 to −7.1)	<.05 (−4.5 to −2.6)	—	<.05 (−8.6 to −6.8)	<.05 (−8.4 to −6.6)	<.05 (−3.7 to −2.06)
rCTL + DNCB (n = 5)	<.05 (9.1 to 10.9)	.586 (−1.1 to 0.64)	<.05 (3.1 to 5)	<.05 (6.8 to 8.6)	—	0.649 (−0.6 to 1.08)	<.05 (3.9 to 5.6)
DNCB + BL (n = 5)	<.05 (8.9 to 10.7)	.319 (−1.3 to 0.44)	<.05 (2.9 to 4.8)	<.05 (6.6 to 8.4)	.649 (−1.08 to 0.6)	—	<.05 (3.7 to 5.4)

Data are presented as *P* value (95% CI).

**Table V:** Eosinophil and mast cell counts, dermal and epidermal thickness, and IgE levels across groups

Groups	Cells		Thickness		IgE ± SD (95% CI)
	Eosinophils ± SD (95% CI)	Mast cells ± SD (95% CI)	Dermal ± SD (95% CI)	Epidermal ± SD (95% CI)	
Only vehicle (n = 5)	1 ± 0.55 (0.52-1.48)	9 ± 1.58 (7.62-10.38)	186 ± 8.12 (178.88-193.12)	29 ± 2.8 (26.55-31.45)	5.43 ± 2.9 (1.8-9.08)
Only DNCB (n = 5)	24 ± 2.34 (21.95-26.4805)	148 ± 8.51 (140.54-155.46)	487 ± 21.2 (468.42-505.58)	154 ± 4.05 (150.45-157.55)	63.4 ± 2.62 (61.1-65.69)
DNCB + topical dexamethasone (n = 5)	2 ± 1.41 (0.62-3. 38)	57 ± 1.14 (56-56)	351 ± 26 (325.52-376.48)	69 ± 2 (67.25-70.75)	15.3 ± 4.14 (8.7-21.9)
DNCB + rCTL (n = 5)	13 ± 2.24 (11.04-14.96)	33 ± 1.01 (135.51-144.49)	339 ± 14 (325.28-352.72)	46 ± 2.6 (43.72-48.28)	18.8 ± 3.8 (14-23.5)
rCTL + DNCB (n = 5)	23 ± 1.58 (21.62-24.38)	140 ± 5.12 (135.51-144. 49)	454 ± 22.7 (434.1-473.9)	129 ± 2.7 (126.63-131.37)	67.5 ± 2.45 (65.35-69.64)
DNCB + BL (n = 5)	22 ± 3.53 (18.91-25.09)	143 ± 2.55 (140.76-145.24)	470 ± 25 (432.09-475.91)	146 ± 1.9 (144.33-147.67)	70.4 ± 1.67 (68.94-71.86)
DNCB + iDEX (n = 5)	8 ± 1.81 (6.41-9.59)	55 ± 2.23 (53.05-56.95)	344 ± 26.89 (320.43-367.57)	68 ± 3.4 (65.02-70.98)	15.7 ± 7.9 (7.4-24.1)

**Fig 2:**
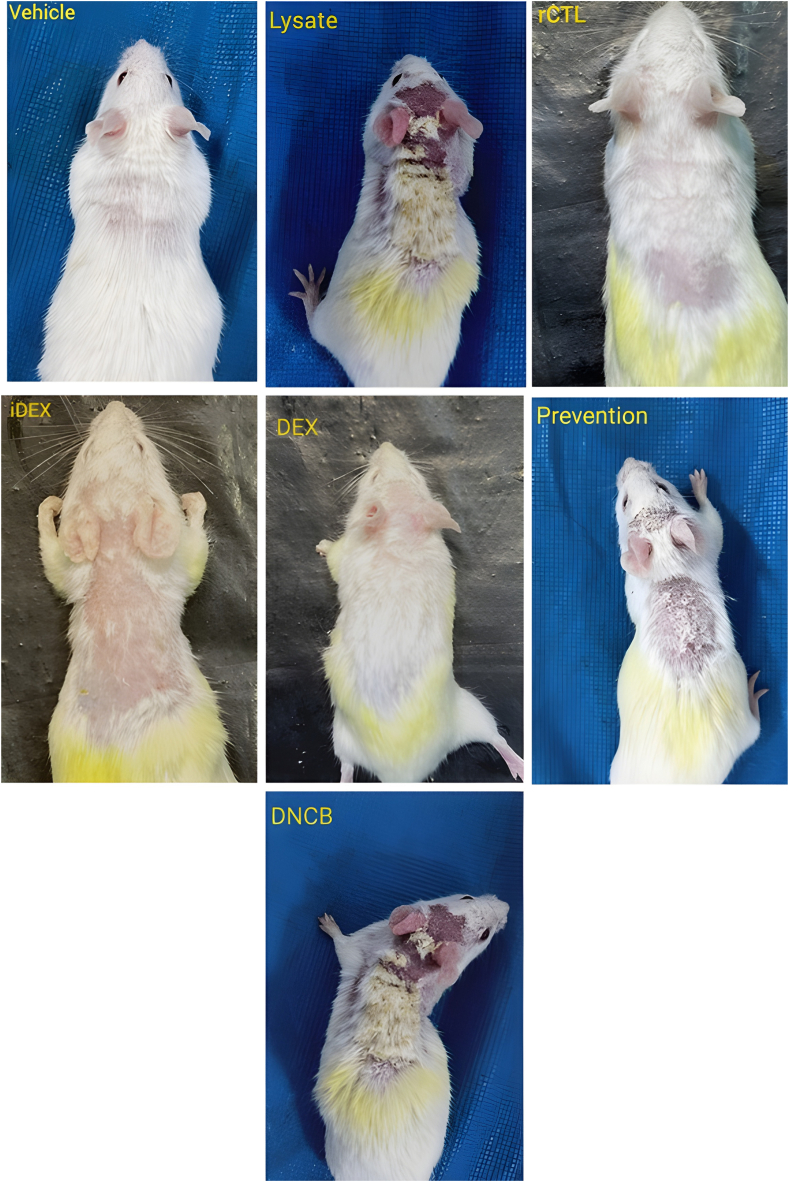
Gross dermatologic changes of the mice in each group at the end of the study. *Top row*, Vehicle: vehicle applied topically with no dermatitis induction; Lysate: dermatitis induced with DNCB, treated with *E coli* BL21 (DE3) lysate administered both subcutaneously and intramuscularly; rCTL: dermatitis induced with DNCB and treated with rCTL administered subcutaneously and intraperitoneally. *Middle row*, iDEX: dermatitis induced with DNCB, treated with dexamethasone administered subcutaneously and intramuscularly; tDEX: dermatitis induced with DNCB and treated with topical dexamethasone; Prevention: pretreated with rCTL before DNCB-induced dermatitis; *Bottom row*, DNCB: dermatitis induced with DNCB, followed by vehicle treatment.

**Fig 3:**
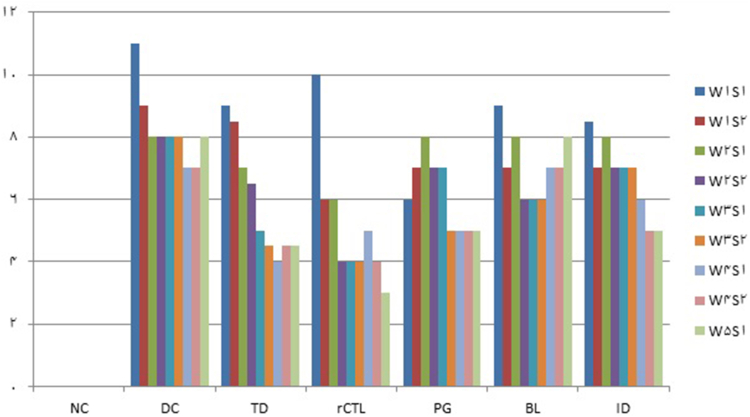
Comparison of changes in the mean dermatitis score across groups over 5 weeks. NC (negative control): only vehicle; DC (DNCB control): only DNCB; tDEX: DNCB + topical dexamethasone; rCTL: DNCB + rCTL; PG: rCTL + DNCB; BL: DNCB + bacterial lysate; iDEX: DNCB + injected dexamethasone. Data were collected twice per week, recorded as W1S1 and W1S2 for week 1, W2S1 and W2S2 for week 2, and so on through week 5 (W5S1).

Fig 2 shows a representative skin section from mice in different groups on the last day of the study. The impact of rCTL and dexamethasone in decreasing mean dermatitis scores during the experiment is shown in Fig 3. The lower dermatitis score observed in the PG group during the first week consistent with the trend in other groups may be attributed to the immunomodulatory effects of rCTL. These effects likely helped attenuate severe inflammatory responses early in the treatment period.

Significant differences in mean pruritic behavior scores were observed between most groups, with the exceptions of the DC group compared with the PG and BL groups, the DC group compared with the iDEX group, and the PG group compared with the BL group, in which no significant differences were detected (Tables III and IV).

**Fig 4:**
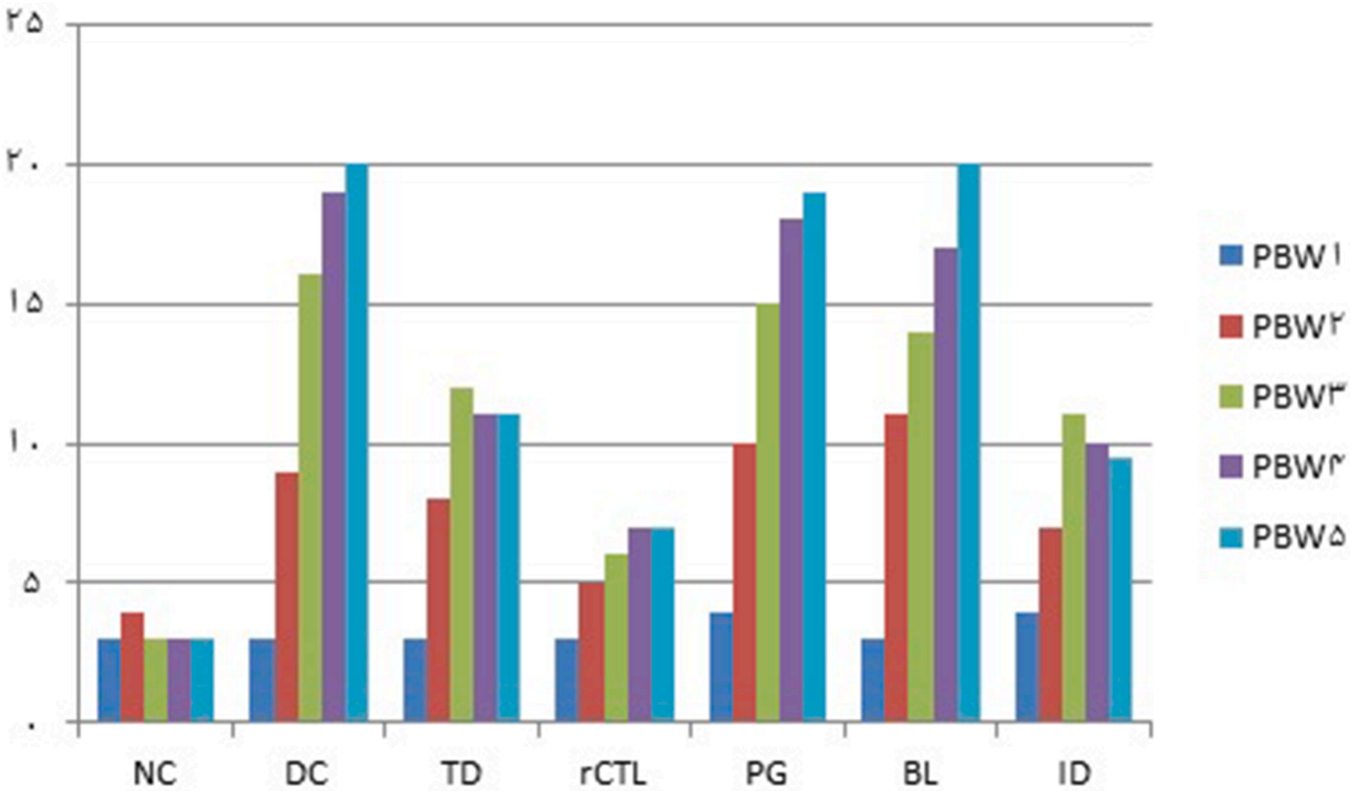
Changes in pruritus behavior score over 5 weeks in different treatment groups. NC (negative control): only vehicle; DC (DNCB control): only DNCB; tDEX: DNCB + topical dexamethasone; rCTL: DNCB + rCTL; PG: rCTL + DNCB; BL: DNCB + bacterial lysate; iDEX: DNCB + injected dexamethasone. *PBW1-PBW5*, Pruritus behavior scores for weeks 1 to 5.

Fig 4 shows the impact of rCTL on the scratching behavior of mice. Following the DNCB challenge, scratching behavior initially increased but gradually declined over the course of the study. Notably, the rCTL group consistently exhibited the lowest pruritus scores across all weeks, demonstrating greater efficacy than dexamethasone in alleviating this clinical sign. In contrast, the PG and BL groups showed pruritus behaviors similar to those observed in the DC group throughout the study period.

The DNCB-challenged mice had significantly increased IgE levels in comparison with the NC group. Also, the IgE levels in rCTL-injected and dexamethasone-treated mice were lower than in the NC group. This number was almost the same in the rCTL and dexamethasone-treated mice (Table V).

**Fig 5:**
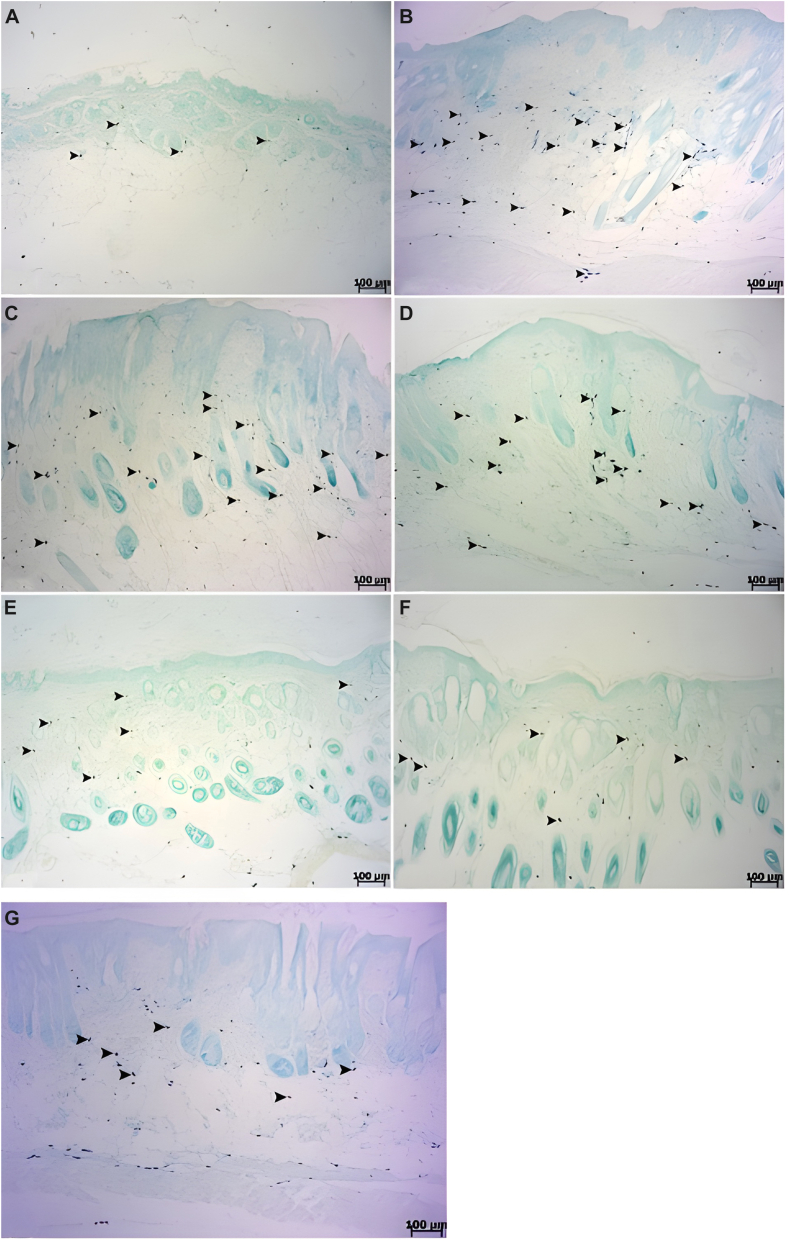
**A-G,** Infiltrated mast cells (*arrowheads*) in the dermis in the only-vehicle group (Fig 5, *A*), only-DNCB group (Fig 5, *B*), PG group (rCTL + DNCB) (Fig 5, *C*), BL group (Fig 5, *D*), tDEX group (Fig 5, *E*), rCTL group (DNCB + rCTL) (Fig 5, *F*), and iDEX group (Fig 5, *G*) (toluidine blue staining).

The skin tissue affected by AD is primarily infiltrated by eosinophils and mast cells, which were assessed in our study using H&E and toluidine blue staining methods (Fig 5). Mice receiving dexamethasone had significantly lower levels of eosinophilic infiltrations in the experimental groups, although greater than in the NC group. Mast cell accumulations in the dorsal skin were most pronounced in the DNCB group, whereas the rCTL group exhibited the lowest number of mast cells, although greater than in the NC group. The tDEX group showed significantly lower levels of mast cell infiltration compared with the DC group, but these levels were still greater than those in the rCTL group. Both the BL and PG groups displayed similar levels of eosinophilic and mast cell infiltration in their skin as observed in the DC group (Tables III and IV).

**Fig 6:**
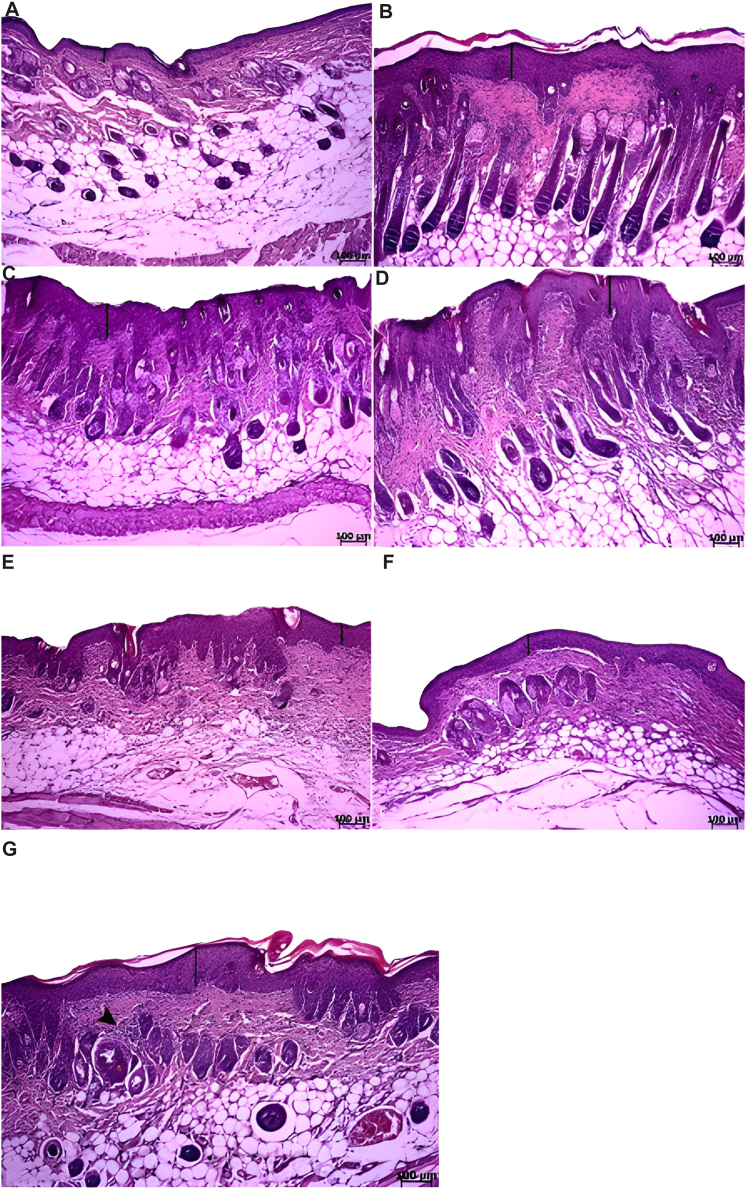
**A-G,** Epidermal thickness (*black lines*) in the NC group (Fig 6, *A*), DC group (Fig 6, *B*), PG group (Fig 6, *C*), BL group (Fig 6, *D*), tDEX group (Fig 6, *E*), rCTL group (Fig 6, *F*), and iDEX group (Fig 6, *G*) (H&E staining).

Repeated applications of DNCB are expected to cause chronic inflammation, resulting in increased thickness of both the epidermis and the dermis. The histopathologic analysis of the present study showed that the DC group exhibited the thickest dermal and epidermal layers, whereas the rCTL group had the thinnest layers (excluding the NC group for comparison). The tDEX group showed lower thickness levels than the DC group but had higher thickness levels than those in the rCTL group. Specifically, the skin tissues in the DC group were 4 times thicker than those of the NC group, and both the BL and PG groups displayed similar thickened skin tissues (Table V) (see also Fig 5 and Fig 6).

ANOVA revealed a significant difference in dermal thickness among the groups (*P* < .05; *F* = 33,179). A *post hoc* Tukey test showed the greatest difference between the only-vehicle group (negative control) and the only-DNCB groups (*Q* = 521.34), whereas the smallest difference was observed between the rCTL and iDEX groups (*Q* = 1.41). Similarly, ANOVA indicated a significant difference in epidermal thickness (*P* < .05; *F* = 5,105), with the Tukey test revealing the largest difference between the only-vehicle group and the DNCB groups (*Q* = 176.7) and the smallest difference between the tDEX and iDEX groups (*Q* = 1.41). Furthermore, ANOVA demonstrated significant differences in IgE levels across groups (*P* < .05; *F* = 2,497.19). The *post hoc* Tukey analysis identified the greatest difference between the only-vehicle group and the DNCB groups (*Q* = 100.4) and the smallest difference between the tDEX and iDEX groups (*Q* = 0.69). Importantly, IgE levels between the tDEX and iDEX groups were not significantly different (*P* = .99; *Q* = 0.69) (Tables III and IV).

## Discussion

This study demonstrated that *T canis* rCTL significantly improved clinical and immunopathologic outcomes in a murine model of atopic-like dermatitis (Figs 1 and 2) (see also Tables III and IV). Among the 7 groups studied, the group treated with rCTL after DNCB induction exhibited the most pronounced reduction in dermatitis severity, with a mean dermatitis score of 3.4 ± 1.14 at week 5, which was significantly lower than both the tDEX and iDEX groups (4.6 ± 0.89) and all other control groups. Notably, the prophylactic administration of rCTL before DNCB challenge resulted in less improvement, indicating that therapeutic intervention during dermatitis induction is more effective. The BL and PG groups showed no significant difference from the DC group, reflecting the specificity of the immunomodulatory effects of rCTL. These findings were further supported by behavioral assessments, wherein the rCTL postinduction group exhibited pruritus scores and a significant reduction in serum IgE compared with controls. Mice in the DC group exhibited the most severe eosinophilic infiltration in their dorsal skin, whereas those treated with rCTL showed the least infiltrations, although still higher than the NC group. Histopathologic analyses also revealed that rCTL treatment markedly decreased epidermal and dermal thickness as well as eosinophil and mast cell infiltration, outperforming dexamethasone. Collectively, these results highlight the therapeutic potential of rCTL in mitigating the clinical and immunologic hallmarks in an AD-like murine model. Further studies are needed to elucidate the discrepancy between the dermatitis and pruritus behavior scores. This could include measuring levels of other cytokines via ELISA and quantifying cell populations such as FoxP3^+^, GATA-3^+^, and T-bet^+^ cells to better understand the underlying mechanisms.

The role of CTLs and CTL-like receptors (CTLRs) and different lectins in dermatitis treatment extends far beyond AD, encompassing a wide range of acute and chronic conditions, including autoimmune diseases, cancer, infectious diseases, and type 1 hypersensitivity.^15^^,^^16^ Primarily expressed on myeloid cells, these receptors serve as crucial regulators of both innate and adaptive immunity by recognizing pathogen-derived and endogenous ligands, thereby modulating immune cell activation, cytokine secretion, and antigen presentation.^17^^,^^18^ In autoimmune diseases, CTLRs have been increasingly recognized for their ability to fine-tune immune responses. For instance, in EAE, a model of multiple sclerosis, *T canis* rCTL alleviates disease severity by promoting anti-inflammatory pathways, such as upregulating FoxP3 expression and enhancing TGF-β production, which support regulatory T-cell function and immune tolerance.^19^ Similarly, dysregulation of CTLRs such as Dectin-3 has been implicated in SLE, wherein targeting these receptors can help restore immune balance by modulating myeloid-derived suppressor cells and T-cell subsets.^20^^,^^21^

CTLRs also play complex roles in sterile inflammation and autoimmune processes, with receptors such as MINCLE capable of either promoting or inhibiting inflammation depending on the ligand and context.^22^^,^^23^ Our findings align with this regulatory function, as administration of rCTL suppressed inflammatory cell infiltration and epidermal hyperplasia in AD, indicating a shift toward immune homeostasis. This immunomodulatory capacity is shared by other CTLR-targeted therapies designed to rebalance dysregulated immune responses.^24^^,^^25^ Moreover, CTLRs exhibit disease-specific immune modulation: in cancer, receptors such as Dectin-1 enhance antitumor immunity by activating proinflammatory cells,^26^ whereas in infectious diseases, they facilitate pathogen recognition and regulate inflammation.^27^, ^28^, ^29^ In contrast, our study demonstrates that rCTL suppresses allergic inflammation in AD by downregulating T_H_2 responses, highlighting the versatile nature of CTLs in either stimulating or dampening immunity depending on the pathologic context.

Histopathologic improvements, including decreased epidermal hyperplasia and dermal inflammatory infiltration, further support the capacity of rCTL to suppress allergic inflammation.^30^^,^^31^ These effects may be mediated through CTL interactions with immune cells such as macrophages and dendritic cells, which recognize parasitic antigens and modulate downstream signaling pathways that control cytokine production and immune cell activation.^32^^,^^33^ The ability of rCTL to reduce T_H_2-driven IgE responses is particularly relevant, because the T_H_2-skewed immune profile is a hallmark of murine AD model pathogenesis.^34^^,^^35^

In our study, treatment with *T canis* rCTL significantly reduced serum IgE levels in the murine model of AD compared with controls other than the normal control. This decrease in IgE, a key mediator of mast cell and basophil activation, indicates that rCTL effectively attenuates the T_H_2-driven immune response characteristic of AD. These findings align with previous observations that *T canis* infection or its components can modulate IgE production and allergic responses, potentially through immune regulatory mechanisms that suppress excessive IgE-mediated inflammation. Thus, the reduction in IgE levels supports the immunomodulatory role of rCTL. Compared with conventional treatments such as dexamethasone, which broadly suppress immune function, rCTL offers a more targeted immunomodulation that may reduce adverse effects associated with long-term steroid use.^36^^,^^37^ This advantage is critical given the chronic nature of AD and the need for safer, disease-modifying therapies.^38^^,^^39^

The findings presented in this study have several important clinical implications with the potential to improve AD management. First, they demonstrate that helminth-derived immunomodulatory molecules represent a fundamentally different therapeutic approach compared with conventional treatments. Unlike current AD therapies that primarily address dermatitis management through corticosteroids and other anti-inflammatory agents, *T canis* rCTL appears to work by modulating underlying immune dysregulation. The superior performance of rCTL compared with that of dexamethasone in reducing dermatitis severity, pruritic behavior, and serum IgE levels suggests that immune tolerance mechanisms induced by helminth-derived molecules may offer advantages over traditional immunosuppression. This is particularly significant given that AD is characterized by a dysregulated T_H_2-dominated immune response, which helminths have evolved to modulate effectively.^16^ The ability of rCTL to simultaneously reduce clinical symptoms (itching and lesions), systemic immune markers (IgE), and histologic evidence of inflammation suggests a coordinated immunoregulatory effect. rCTL data reveal a precise mechanism capable of contributing to the modified type-2 immunity.

The observed histopathologic enhancements, specifically a reduction in epidermal thickening and inflammatory cell infiltration, demonstrate that rCTL induces significant structural alterations in the affected tissue, going beyond simple symptom alleviation. This distinction is clinically important because it suggests the potential for long-term disease modification. These findings are further supported by observations in the EAE mouse model treated with rCTL, in which infiltration of mononuclear cells significantly decreased compared with the control group (in which EAE was induced without treatment),^15^ along with a concurrent increase in the FoxP3-positive cell population.^24^

Helminth-derived secretory products, particularly glycoconjugate-rich molecules such as CTLs, represent an untapped reservoir of immunomodulatory compounds with therapeutic potential. Rather than using broad immunosuppression, these molecules work through the parasite’s refined mechanisms of immune evasion, which are optimized over millions of years of coevolution with mammalian hosts.^12^ This evolutionary perspective suggests that such molecules may induce more physiologic and sustainable immune tolerance than synthetic immunosuppressants. Because helminth secretory products can influence multiple immune pathways by binding to specific receptors,^16^ a therapy using these substances is likely to be more effective than treatments targeting only 1 pathway. The study’s finding that rCTL outperformed dexamethasone, a potent corticosteroid effective for AD, across multiple outcome measures (dermatitis severity, pruritic behavior, serum IgE, and histologic markers) is clinically significant. This superiority suggests that immunomodulation through helminth-derived molecules may represent a more effective therapeutic strategy than conventional anti-inflammatory approaches.

Although these results are promising, subsequent research must identify the specific receptors and signaling pathways to fully elucidate the molecular mechanisms of *T canis* CTL. Long-term safety and efficacy evaluations in diverse models, and eventual clinical trials, may help to validate the translational potential of rCTL for human AD treatment. In addition, exploring the broader applicability of parasitic CTLs in other allergic and autoimmune conditions may open new avenues for immunotherapy.

This study provides strong evidence that *T canis* rCTL exerts a powerful immunomodulatory effect that significantly alleviates both the clinical manifestations and underlying immunopathology of AD in a murine model, and these require comparison with conventional treatments such as dexamethasone. rCTL not only reduced dermatitis severity and pruritus more effectively but also demonstrated superior suppression of serum IgE levels and inflammatory cell infiltration in the skin. These findings suggest that rCTL targets key immune pathways involved in AD pathogenesis, offering a promising novel therapeutic strategy that goes beyond symptom relief to address disease mechanisms. Although further research is needed to elucidate the precise molecular mechanisms, optimize dosing, and evaluate long-term safety and efficacy, the present results lay a solid foundation for the development of helminth-derived molecules such as rCTL as innovative immunotherapies for AD-like murine model. Ultimately, such advances could transform this condition, improving patient outcomes and quality of life.

Data availability: Data sets generated during the present study are available from the corresponding author on request.

Ethics: This study was conducted in accordance with the ethical guidelines and principles outlined by the rules for working with laboratory animals at the University of Tehran. The study protocol was approved by the university’s ethics committee under the code IR.UT.VETMED.REC.1403.010.

## Disclosure statement

This study was funded by the 10.13039/501100003968Iran National Science Foundation (grant no. 4003141), and additional funding was obtained from the Faculty of Veterinary Medicine, Ministry of Research, University of Tehran (grant no. 7508043-6-71).

Disclosure of potential conflict of interest: The authors declare that they have no relevant conflicts of interest.

## References

[1] Otsuka A., Nomura T., Rerknimitr P., Seidel J.A., Honda T., Kabashima K. (2017). The interplay between genetic and environmental factors in the pathogenesis of atopic dermatitis. Immunol Rev.

[2] Darabi B., Rizehbandi M., Shokri M., Shokri F., Bastani E. (2023). Prevalence of pediatric atopic dermatitis in Iran: a systematic review and meta-analysis. Int J Pediatr.

[3] Safiri S., Jaberinezhad M., Mousavi S.E., Motlagh Asghari K., Shamekh A., Nejadghaderi S.A. (2024). The burden of dermatitis from 1990–2019 in the Middle East and North Africa region. BMC Public Health.

[4] Narla S., Silverberg J.I. (2020). The role of environmental exposures in atopic dermatitis. Curr Allergy Asthma Rep.

[5] Drechsler Y., Dong C., Clark D.E., Kaur G. (2024). Canine atopic dermatitis: prevalence, impact, and management strategies. Vet Med (Auckl).

[6] Marsella R. (2021). Advances in our understanding of canine atopic dermatitis. Vet Dermatol.

[7] Li H., Zhang Z., Zhang H., Guo Y., Yao Z. (2021). Update on the pathogenesis and therapy of atopic dermatitis. Clin Rev Allergy Immunol.

[8] Arora C.J., Khattak F.A., Yousafzai M.T., Ibitoye B.M., Shumack S. (2020). The effectiveness of Janus kinase inhibitors in treating atopic dermatitis: a systematic review and meta-analysis. Dermatol Ther.

[9] Gonzales A.J., Aleo M., Mahabir S., Messamore J., Stegemann M. (2024). Oclacitinib (APOQUEL®) is a selective Janus kinase 1 inhibitor with efficacy in a canine model of flea allergic dermatitis. J Vet Pharmacol Ther.

[10] Tsai S.Y., Phipatanakul W., Hawryluk E.B., Oyoshi M.K., Schneider L.C., Ma K.S. (2024). Comparative safety of oral Janus kinase inhibitors versus dupilumab in patients with atopic dermatitis: a population-based cohort study. J Allergy Clin Immunol.

[11] Wojciechowski D., Wiseman A. (2021). Long-term immunosuppression management: opportunities and uncertainties. Clin J Am Soc Nephrol.

[12] Etebar F., Hosseini S.H., Jalousian F., Ranjbar M.M. (2018). Phylogenetic analysis of C type lectin from *Toxocara canis* infective larvae and comparison with the C type lectin family in the immune system of mouse and human. Iran J Parasitol.

[13] Malekzadeh P., Hosseini S.H., Shahbakhsh M., Shayan P., Zibaei M., Jamshidi S. (2024). Preliminary study of *Toxocara canis* recombinant C-type lectin as a suitable antigen for serodiagnosis of human toxocariasis. J Zoonotic Dis.

[14] Charan J., Kantharia N. (2013). How to calculate sample size in animal studies?. J Pharmacol Pharmacother.

[15] Shahbakhsh M., Jalousian F., Hosseini S.H., Moghadasi A.N., Shayan P., Bonab S.F. (2025). *Toxocara canis*-originated recombinant C-type lectin improves the disability scores of experimental autoimmune encephalomyelitis in murine in vivo models. J Neuroimmunol.

[16] Etebar F., Hosseini S.H., Borhani Zarandi M., Moghadasi A.N., Jalousian F. (2023). The immunomodulatory effects of the C-type lectin protein of Toxocara canis on experimental autoimmune encephalomyelitis. Parasite Immunol.

[17] Jang S., Ohn J., Kim J.W., Kang S.M., Jeon D., Heo C.Y. (2020). Caffeoyl–pro–his amide relieve DNCB-induced atopic dermatitis-like phenotypes in BALB/c mice. Sci Rep.

[18] Riedl R., Kühn A., Hupfer Y., Hebecker B., Peltner L.K., Jordan P.M. (2024). Characterization of different inflammatory skin conditions in a mouse model of DNCB-induced atopic dermatitis. Inflammation.

[19] Laske N., Niggemann B. (2004). Does the severity of atopic dermatitis correlate with serum IgE levels?. Pediatr Allergy Immunol.

[20] Geijtenbeek T.B., Gringhuis S.I. (2016). C-type lectin receptors in the control of T helper cell differentiation. Nat Rev Immunol.

[21] Salazar F., Sewell H.F., Shakib F., Ghaemmaghami A.M. (2013). The role of lectins in allergic sensitization and allergic disease. J Allergy Clin Immunol.

[22] Saijo S., Iwakura Y. (2011). Dectin-1 and Dectin-2 in innate immunity against fungi. Int Immunol.

[23] Goodridge H.S., Wolf A.J., Underhill D.M. (2009). Beta-glucan recognition by the innate immune system. Immunol Rev.

[24] Shahbakhsh M., Hosseini S.H., Bonab S.F., Malekzadeh P., Moghadasi A.N., Shayan P. (2022). Recombinant C-type lectin protein of Toxocara canis increases the population of spleen Foxp3+ regulatory T cells in BALB/c mice. Ann Parasitol.

[25] Wang T., Pan D., Zhou Z., You Y., Jiang C., Zhao X. (2016). Dectin-3 deficiency promotes colitis development due to impaired antifungal innate immune responses in the gut. PLoS Pathog.

[26] Alves I., Fernandes Â., Santos-Pereira B., Azevedo C.M., Pinho S.S. (2022). Glycans as a key factor in self and nonself discrimination: impact on the breach of immune tolerance. FEBS Lett.

[27] Wells C.A., Salvage-Jones J.A., Li X., Hitchens K., Butcher S., Murray R.Z. (2008). The macrophage-inducible C-type lectin, mincle, is an essential component of the innate immune response to *Candida albicans*. J Immunol.

[28] Yamasaki S., Ishikawa E., Sakuma M., Hara H., Ogata K., Saito T. (2008). Mincle is an ITAM-coupled activating receptor that senses damaged cells. Nat Immunol.

[29] van Kooyk Y., Ilarregui J.M., van Vliet S.J. (2015). Novel insights into the immunomodulatory role of the dendritic cell and macrophage-expressed C-type lectin MGL. Immunobiology.

[30] Dennehy K.M., Brown G.D. (2007). The role of the β-glucan receptor Dectin-1 in control of fungal infection. J Leukoc Biol.

[31] Cheng S.C., van de Veerdonk F.L., Lenardon M., Stoffels M., Plantinga T., Smeekens S. (2011). The dectin-1/inflammasome pathway is responsible for the induction of protective T-helper 17 responses that discriminate between yeasts and hyphae of *Candida albicans*. J Leukoc Biol.

[32] Robinson M.J., Osorio F., Rosas M., Freitas R.P., Schweighoffer E., Gross O. (2009). Dectin-2 is a Syk-coupled pattern recognition receptor crucial for Th17 responses to fungal infection. J Exp Med.

[33] Angelina A., Martín-Cruz L., de la Rocha-Muñoz A., Lavín-Plaza B., Palomares O. (2023). C-type lectin receptor mediated modulation of T2 immune responses to allergens. Curr Allergy Asthma Rep.

[34] Rabinovich G.A., Croci D.O. (2012). Regulatory circuits mediated by lectin-glycan interactions in autoimmunity and cancer. Immunity.

[35] Novak N., Bieber T. (2003). Allergic and atopic skin diseases. J Allergy Clin Immunol.

[36] Brandt E.B., Sivaprasad U. (2011). Th2 cytokines and atopic dermatitis. J Clin Cell Immunol.

[37] Allen J.E., Maizels R.M. (2011). Diversity and dialogue in immunity to helminths. Nat Rev Immunol.

[38] Gause W.C., Rothlin C., Loke P. (2020). Heterogeneity in the initiation, development and function of type 2 immunity. Nat Rev Immunol.

[39] Bieber T. (2008). Atopic dermatitis. N Engl J Med.

